# The use of social prescribing and community-based wellbeing activities as a potential prevention and early intervention pathway to improve adolescent emotional and social development: a systematic mapping review

**DOI:** 10.1186/s12889-025-24413-5

**Published:** 2025-10-15

**Authors:** Siobhan B. Mitchell, Lucy Cartwright, Alex Gude, Alison Bethel, Harriet Hunt, Jane R. Smith, Daniel Hayes, Marcello Bertotti, Paul Jarvis-Beesley, Vashti Berry, Kerryn Husk

**Affiliations:** 1 University of Exeter Medical School, University of Exeter, St. Luke’s Campus, Heavitree Road, Exeter, EX1 2LU UK; 2 NIHR ARC South West Peninsula (PenARC), Faculty of Health, University of Plymouth, Plymouth, UK; 3 Evidence Based Practice Unit (EBPU), University College London and the Anna Freud National Centre for Children and Families, London, England; 4 Institute for Connected Communities, University of East London, London, England; 5 Social Prescribing Youth Network (SPYN), Streetgames, Manchester, England

**Keywords:** Children, Young people, Social prescribing, Community, Mapping

## Abstract

**Background:**

Social prescribing (SP) initiatives which aim to connect individuals to community-based assets have received increased research and policy attention, however schemes have mostly centred on adults. There is little research examining how social prescribing might work for children and young people (CYP), and specifically, what pathways into social prescribing look like for this group.

**Methods:**

We conducted a systematic mapping review to understand what social prescribing pathways look like for CYP. Searches were carried out in February 2022. We reviewed published journal articles and grey literature on pathways for CYP accessing activities or services in the community. We synthesised studies through tabulation and narrative descriptions of similarities and differences.

**Results:**

We identified 14,518 unique hits through electronic database searches, and an additional 230 through grey literature searches. Following exclusions at title/abstract and full text stage, a total of 35 articles from the database searches and 33 sources from the grey literature search were included in the review.

**Discussion and conclusion:**

Included papers described a broad range of approaches, cohorts, implementation, and roles included in services. Pathways into SP for CYP seem to vary from the adult SP model, which primarily utilises a primary care pathway, with referrals for CYP SP mostly through educational institutions but also through GPs and self-referral. Link workers or another ‘linking function’ was often (but not always) involved in the pathway. Future research should examine how and in what ways particular cohorts access, or not, these sorts of pathways, the economic impact, and examine any potential risks or harms that might be associated with CYP-SP. Funding: National Institute for Health and Care Research (NIHR) School for Primary Care Research (project reference MH003).

**Trial registration:**

PROSPERO 2022 CRD42022312745.

**Supplementary Information:**

The online version contains supplementary material available at 10.1186/s12889-025-24413-5.

## Background

There has been a rise in young people’s mental health conditions observed in both primary and secondary care and in the community, with specialist services struggling to meet demand, particularly in the wake of the COVID-19 pandemic [37, 39]. While social prescribing initiatives have received increased research and policy attention as a means of improving people’s mental health, schemes have mostly centred on adults [4].

Adolescence is a period of marked physical, emotional and social change in which the fostering of social connectedness outside of the immediate family unit is core to identity development and mental wellbeing. Research indicates that three quarters of mental health difficulties occur before the age of 25 and half before age 14 [23]. However, current statutory services are not geared towards preventing these difficulties and struggle to provide early intervention, meaning many young people do not get the help and support needed at a critical time, leaving problems unresolved. Even when help and support are eventually provided, this usually follows multiple help-seeking contacts and lengthy waits for statutory services [11].

There is a limited but growing evidence-base linking social connectedness and community participation to young people’s mental health and wellbeing [24, 46]. However, little is known about how young people themselves understand these concepts,what opportunities for connectedness and participation exist outside of school settings; or how, and to what extent, such opportunities impact on young people’s healthy emotional and social development. Furthermore, very little evidence, policy and provision around promising social prescribing initiatives—which aim to connect individuals to community-based assets and activities relevant to their interests and goals—have applied specifically to children and young people (CYP), though there are calls for this. For example, a recent Wellcome report advocates for ‘young people and communities driving innovation in mental health’ [44].

Social prescribing is gaining traction globally, but with differential and locally driven implementation in each country [28]. For example, in the UK there has been widespread adoption of social prescribing as an idea, but this has been interpreted differently across the devolved nations: In Wales, the model is more community and public-health focused,in Scotland there is a more primary care centric model but without universal funding,while England has systematised GP-centred link working as a core health role that is funded through core health policy [28]. Research on social prescribing for CYP is in its infancy, with a recent scoping review concluding that while existing evidence is sparse, outcomes are promising,with several evaluations suggesting increases in mental, physical and social health and reduction in healthcare demand [29].

Social prescribing for adults has been conceptualised as “…the patient pathway from primary care to whichever activity undertaken” [19], p310) and it has been acknowledged that this pathway can take multiple forms. Examples of this include signposting from primary care directly to the activity e.g., through a leaflet,direct clinician referral to an activity (e.g., exercise on referral),link worker referral using a linking function, such as referral from a primary care clinician to a link worker and then signposted to an activity; or a holistic model where the person is directly and flexibly supported (via one or more sessions at the discretion of the link worker) with both health and social needs. The latter two models are the models now financed and supported through core government policy in England, with every general practice now having access to a social prescribing link worker [36]. A focus of this review is whether these models or pathways for social prescribing are used, are suitable, or need to be adapted for CYP.

Socially prescribed activities for adults can be categorised into four main domains or pillars [31]. The four pillars include advice and information (e.g., practical help with financial and legal problems), arts and heritage (e.g., taking an art class or volunteering at a local museum), natural environment (e.g., joining a local gardening or food-growing project), and physical activity (e.g., learning to dance or joining a cycling club) [31]. Whether socially prescribed activities for CYP can be categorised in the same way has not yet been explored and so our review will also assess this.

Pathways to care are defined by the help-seeking behaviour of the individual, the sequence of contacts the individual has with statutory services and other informal contacts and non-statutory services such as charities, and how those respond to their needs. A recent review exploring pathways for young people with mental health difficulties identified that the number of contacts ranged from 0 to 15 (with a pooled mean of 2.9 contacts) per participant, covering a wide range of professional and non-professional contacts [25]. Population studies suggest that informal contacts such as teachers or other education staff are the most common sources of help and advice for children aged 8–16 years [34, 40, 43]. In the MHCYP 2022 survey children were asked directly about accessing MH support in school with 1 in 4 (25.1%) 11–16 year olds reporting accessing mental health and wellbeing support at school in the past year and 59.8% of children with a probable mental health disorder reporting accessing support in this way [33].

Common barriers to accessing support include stigma, service demand, and lack of trust [2, 15]. Pathways into activities and statutory services are also known to be affected by factors such as age, gender and ethnicity, deprivation and presenting difficulty [42]. For example, research shows that ethnicity plays an important role in explaining how young people access mental health services, with Black and mixed-race young people being more than twice as likely to be referred through social care/youth justice than primary care when compared to their white counterparts [6, 22].

In the UK, where social prescribing has been widely adopted, identification of, and support for, young people with mental health difficulties remains complex and multifaceted. Schools are rightly seen as an important setting in which to identify and treat mental health difficulties, with the UK Department for Education running one of the largest randomised trials to explore how to improve mental health in schools [9]. Voluntary and community organisations are also playing an increasing role in mental health support, including via the incorporation of newer initiatives such as social prescribing [36]

Understanding the ways in which young people approach and access mental health support is pivotal to the development of appropriate person- or family-centred care, including social prescriptions. This includes building pathways and services which are accessible, facilitate choice, and provide support in a timely way at various stages of children’s and young people’s development [12]. In light of the recent pandemic, which has impacted upon demand and availability of services, understanding how pathways to care may need to be adapted is also an important consideration [27, 41]. The aim of this review was to explore how social prescribing schemes/pathways operate as a vehicle for assisting CYP with mental health difficulties to access, connect with and participate in community activities.

## Methods

In this review, we sought to understand the pathways for CYP accessing activities or organisations in the community; who the first point of contact is; what happens next in this process; and what are the ‘touch points’ along the way (i.e., the pathway and/or linking function after this initial point of contact to link them into social prescribing/community assets and activities). In doing so, this will allow us to consider how and where social prescribing might be positioned to be of most use to CYP in need. We were also interested to explore whether there were differences in these findings based on demographic and clinical characteristics of those seeking support.

We undertook a systematic mapping review of published journal articles and grey literature on pathways for CYP accessing activities or organisations in the community i.e., akin to social prescribing though not necessarily described as such, for their mental health and wellbeing. For the complete review protocol, please see Mitchell et al. [26]. As with other mapping reviews [5], we did not conduct formal quality appraisal. We synthesised studies through tabulation, juxtaposition and narrative descriptions of similarities and differences. Our aim was not to assess the effectiveness of particular approaches as we know the data are limited and pathways/activities heterogenous. Systematic Maps do not aim to answer a specific question, but instead collate, describe and map findings in terms of distribution and abundance of evidence, often configured in relation to different elements of a question [14, 20].

### Inclusion and exclusion criteria (Table 1)

**Table 1:** Inclusion and exclusion criteria

Inclusion criteria	Exclusion criteria
Describing direct work with CYP up to age 25 i.e., the primary target should be the child or young person as opposed to a parent or carer	Primary target adult rather than CYP population
Any study or source describing a pathway to community (VCSE, not statutory) activities for CYP aged < 25 years. This included CYP self-referring, where this was clearly described We define a community asset as an activity that is hosted by the voluntary and community sector, attended by CYP voluntarily, and centred around activities from one or more of the four ‘pillars’ of social prescribing: Physical activity, arts and heritage, natural environment, advice and information – and other children’s activities	• Activity only described with no pathway • Evidence of a pathway missing or unclear • Community asset missing or unclear • Experimental service rather than real-life • Clinical setting (as opposed to community setting)
Population experiencing or at risk of mental health issues	Universal offer
Published in English between 1999 and 2022	

#### Database search strategy

We searched the following databases between 15–17 Feb 2022: MEDLINE (1946-), APA PsycINFO (1806-), Social Policy and Practice (1890-), Healthcare Management Information Consortium (1979-) (via Ovid), Web of Science Core Collection: Science Citation Index (1990-), Social Science Citation Index (1990-), Arts and Humanities Citation Index (1975-), Conference Proceedings Citation Index – Science (1990-), Conference Proceedings Citation Index – Social Science and Humanities (1990-), Emerging Sources Citation Index (2015-) (Clarivate), British Nursing Database (1993-), ProQuest Dissertations & Theses Global (1637-)(ProQuest), CINAHL Complete (1937-), Allied and Complementary Medicine Database, Sociology Collection which included ASSIA (1937-_(via EBSCOhost), Scopus (1788-) (Elsevier), Epistemonikos (no dates available) (https://www.epistemonikos.org/en/) and the Cochrane Library both CDSR (1996-) and Central (1908-) (Wiley). An additional search was undertaken in Google Scholar using Hartzing’s Publish or Perish and another, very precise search, in AEI (1977-), Education Collection (1966-), Humanities Index (1962-), International Bibliography of the Social Sciences (1965-), on ProQuest and British Education Index, Child Development and Adolescent Studies, Education Abstracts, Education Research Complete, Education Resources Information Center, Humanities International Complete, Psychology and Behavioural Sciences Collection in EBSCOhost. Forwards and backwards citation searching was carried out in November 2022 out of the 35 included papers from the database screening using Scopus, Web of Science or Google Scholar. For Medline search strategy, see Appendix a.

#### Grey literature search strategy

In line with other reviews on similar topics we conducted additional searches for grey literature [7, 8]. We completed web searching using Google and two sets of search terms: “social prescribing link worker” and “young people social prescribing mental health”. All results pages were screened for each search. We also searched a set of specific websites drawn together from a Call for Evidence we sent out to networks and organisations and from social prescribing services (see Appendix b).

#### Screening titles and abstracts

Titles and abstracts were double- and blind-screened in Rayyan by two researchers (SM, LC, AG) using the inclusion/exclusion criteria set out above. Discussion with a third researcher (KH, JS) took place for any complex or conflicting decisions.

#### Screening full texts

**Fig. 1:**
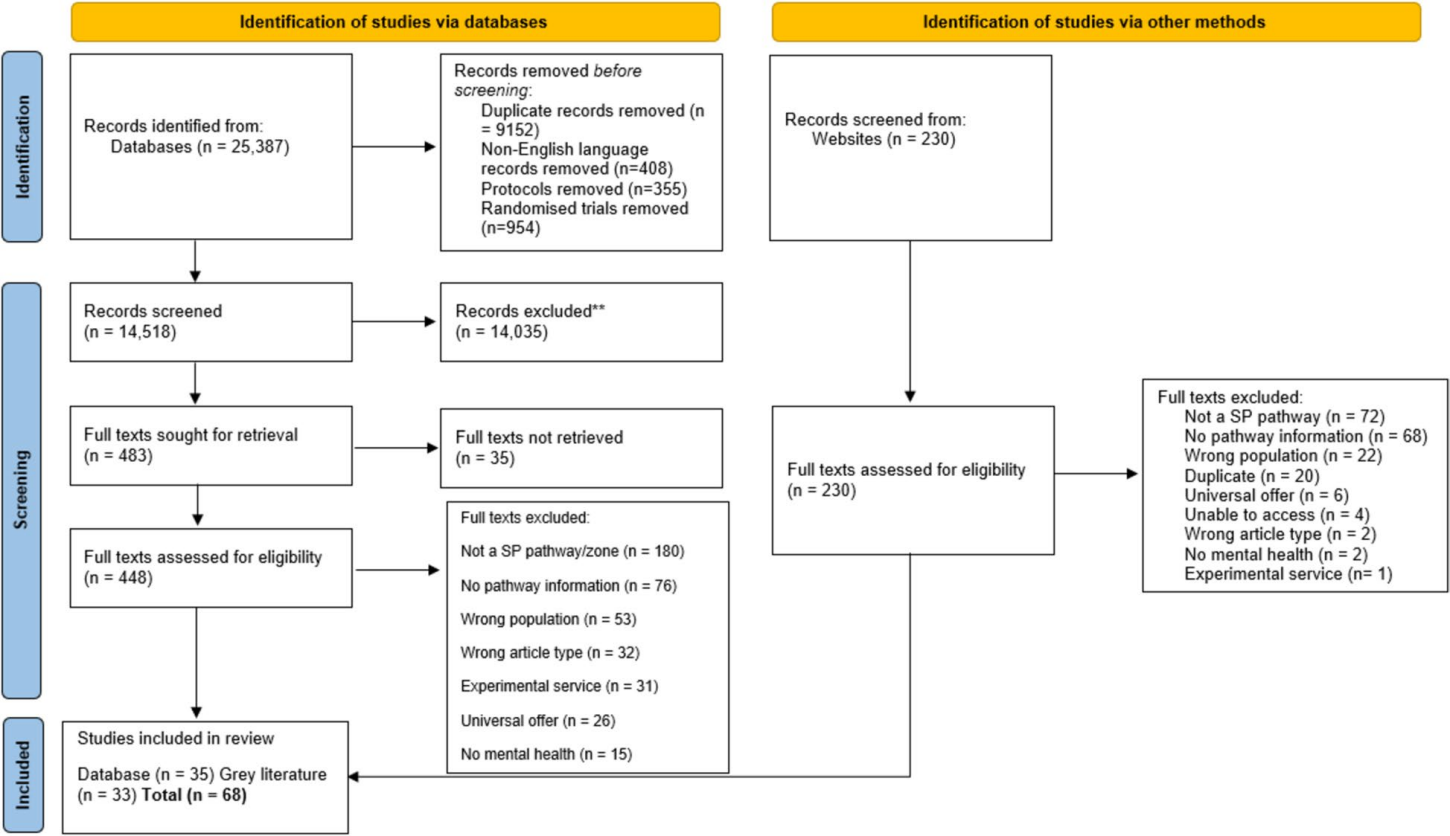
PRISMA diagram. *From:* Page MJ, McKenzie JE, Bossuyt PM, Boutron I, Hoffmann TC, Mulrow CD, et al. The PRISMA 2020 statement: an updated guideline for reporting systematic reviews. BMJ 2021;372:n71. https://doi.org/10.1136/bmj.n71. For more information, visit: http://www.prisma-statement.org/

Full-texts were also double-screened in a separate Rayyan file by two researchers (SM, LC, AG), with additional discussion involving the wider team and/or a third researcher (KH, JS). The most common reasons for exclusion were that the study did not meet our criteria for reporting a social prescribing pathway (as described above, e.g., no community asset) or there was no pathway information provided (see Fig. 1).

#### Data extraction

Data were extracted into bespoke Excel spreadsheets and included location of social prescribing service (i.e., country), delivery setting (e.g., school, youth centre), description of population (i.e., age or other eligibility criteria), aim of study, outcomes assessed, referral source (e.g., school, parent, GP), linking function (i.e., how CYP get from initial contact to activity), nature of activity and duration of engagement, and reason for participant referral. The National Academy of Social Prescribing’s four pillars (Advice and Information, Arts and Heritage, Natural Environment and Physical Activity) were used as a framework for grouping activities [31]. Extraction of data at full text was completed across three researchers (SM, LC, AG) who worked independently in this task. Study investigators were not contacted if there were any missing data, but missing data were recorded. Any unclear data were discussed between these three researchers to reduce risk of bias, and then recorded as unclear if agreed.

#### Data synthesis

Extracted data were synthesised iteratively by the whole review team through a series of discussion meetings. Each study was read by at least two reviewers, and discussions held to review findings and establish areas of similarity and difference. Pathway and individual paper characteristics have been tabulated and are presented here, alongside narrative descriptions of social prescribing services and linking elements.

## Results

We identified 14,518 unique hits through electronic database searches, and an additional 230 through grey literature searches. Following exclusions at title/abstract and full text stage, a total of 35 articles from the database searches and 33 sources from the grey literature search were included in the review (see Fig. 1).

We will discuss findings by service. We define a social prescribing service as a set of organisations that collaborate to deliver a social prescribing pathway. Within this, there are social prescribing organisations i.e., the entities that deliver social prescribing and can include a VCSFE or third sector organisation that delivers an activity, a referral organisation such as a school or GP practice, or a brokering organisation such as a charity.

Supplementary Table 2 details included services and their delivery setting and location; age of CYP served and other eligibility criteria; and reasons for referral to the service.

### Social prescribing services

Of the 68 sources included in this review, 72 separate social prescribing services were described (some sources included more than one organisation). Four organisations appeared more than once in our inclusions: Wave Project, Tackling the Blues, Aberdeen Foyer and Headspace. Where possible, we have not included duplicated descriptions, although some sources provided additional details which are incorporated. In Supplementary Table 2, where there is a reference to the same service but in a separate study, references have been organised to group studies pertaining to the same service together.

### Setting for delivery

Settings for delivery varied widely, with many activities also having more than one delivery setting e.g., at school, after school, and at home. Settings included youth or community centres/clubhouses (*N* = 17), schools (*N* = 15), youth mental health service (*N* = 5), home (*N* = 4), community or wellbeing cafes (*N* = 4), and local parks/woodland, beach, farm, hospital/emergency department, local football club, youth homeless centre, charity head office, online (< *N* = 3 each).

Twenty-three of the 72 separate social prescribing services did not specify a delivery setting.

### Location

With the caveat that only studies reported in English were included, a wide range of locations were covered; with studies from across 10 different countries included and the majority stemming from England (*N* = 44, plus 2 UK-wide). Others included were from Scotland (*N* = 5), Wales (*N* = 1), Australia (*N* = 7), Canada (*N* = 5), United States (*N* = 4), and Finland, Israel, Jamaica, and New Zealand (*N* = 1 each).

### Age and other participant characteristics

The age of CYP in included studies also varied widely and was influenced by the setting for delivery e.g., with younger ages catered to by school delivery settings. Five studies did not specify an age range but described the population in more broad terms such as ‘young adults’, ‘youth’, and ‘young people’. Where reported, ages served by services ranged from 4–29 years, with the age range sometimes being extended beyond age 24 based on specific needs e.g., SEND. The most common age ranges reported were 16–24 or 25 years (*N* = 8), 11–25 or 26 years (*N* = 6), and 14–25 years (*N* = 5). The most common age for services to start their offer was 11 years (*N* = 8) and to end their offer was 24 or 25 years (*N* = 11) or 16 years (*N* = 8).

Other criteria for accessing services were geographical including being registered at a specific GP practice, attending a school in a specific Primary Care Network or living in a particular area (*N* = 7). Two specified criteria related to sex (1 male only, 1 female only), and single studies specified not being in urgent care or with Child & Adolescent Mental Health Services (CAMHS), attendance at a youth centre, current involvement with the service, and acknowledgement of and seeking help for a mental health problem.

### Reason for referral

Five (out of 68) sources did not state a reason for referral but where stated, reasons could be multiple, were very diverse and included, in order of frequency:

- *Social and relational issues* (*N* = 41) including loneliness, family and relationship issues, social isolation (or risk of), bullying, attachment issues, at risk due to mixing with known offenders, involvement in crime/criminal justice system, at risk in the community, social exclusion, and lacking in confidence to make friends/difficulty with social integration.
- *General mental health and wellbeing concerns* (*N* = 15) and CYP identified as having *low to moderate mental health needs or being at risk* (*N* = 17); most commonly, emotional problems, anxiety/excessive worrying, depression and low mood, stress, lack of confidence and/or self-esteem, self-harm behaviours. Other reasons included bereavement, body image and eating issues, and anger.
- *General or physical health and wellbeing problems* (*N* = 15) including with sleep, exercise, sexual health, and need for support with long-term health conditions.
- *Socioeconomic challenges* (*N* = 15) such as hardship, money worries or poverty, being at risk of or experiencing homelessness, and occupational or work difficulties.
- *School support* (*N* = 12) such as attendance issues/barriers to attendance, education issues, and exam stress.
- *Severe mental health issues* (*N* = 8) such as psychosis, clinical diagnosis of at-risk mental health state (ARMS), psychiatric hospitalisation, psychotic-like experiences in addition to other significant psychopathology, attending an emergency department in crisis.
- *Transition support* (*N* = 8) including recovery from mental health problems, leaving care, transitioning from child to adult mental health services, starting at a new school, and parental separation.
- *Substance use and addiction* (*N* = 7).
- *Involvement with other services* (*N* = 3) including social services, being in receipt of other support services, or on a waiting list for other services such as CAMHS.

In relation to mental health and wellbeing, many sources used only ‘mental health’ or ‘mental health issues’ to describe this, and we are therefore unable to report on specific mental health issues for these sources. Others used the terms ‘emotional wellbeing/problems/distress/difficulties/support’.

While the majority of descriptions of reasons for referral did not include specification around the severity of mental health issues, some sources described serving populations who were perceived to be ‘facing or at risk’ of mental health issues; those with ‘low level’ or ‘low to moderate’ mental health needs; those who may be ‘falling between gaps of services’; and those who displayed ‘symptoms or behaviours associated with poor mental health which might lead to diagnosis if accessing specialist mental health services’. Some mental health reasons for referral were described as diagnosed mental health needs or being of clinical severity.

### Outcomes assessed

Whilst this is not a review of effectiveness, and so we do not synthesise results, outcomes assessed in the included studies were also mapped [30]. Twenty-two of the included studies reported use of quantitative outcome measures. Outcomes related to psychological wellbeing (reported in 20 out of the 22 studies with this data) and social wellbeing (reported in 13 out of 22 studies) were the most frequently reported. Outcomes related to psychological wellbeing ranged from broad measures of mental or emotional wellbeing or mood, to specific measures of problem severity/symptom reduction e.g., depression, self-esteem, self-confidence, resilience, sense of mastery, autonomy, or global functioning. Outcomes related to social wellbeing and skills included measures of loneliness, social wellbeing, social trust, social comfort, social capital, social skills and relationships, social competence, social inclusion or connection, interpersonal skills, and social engagement.

Other outcomes related to behaviour/delinquency (*N* = 6), physical activity and health (*N* = 4), school attendance and attainment (*N* = 4), use of services (*N* = 3) (e.g., GP consultations, Accident & Emergency attendance, hospital admission), connectedness to nature (*N* = 1), engagement in further education and training (*N* = 1), stability of housing (*N* = 1), and family functioning (*N* = 1).

The impact on outcomes was reported to be largely positive (18 studies out of 22) with outcomes such as mental wellbeing, use of services, and loneliness showing improvement after CYP engagement in community activities.

### Pathway data

Supplementary Table 3 reports information relating to pathways for CYP accessing social prescribing services. This information is organised around a standard social prescribing model [18] and includes information about who refers CYP to social prescribing services,how they are linked to community activities and if this includes a ‘linking function’ or ‘linking role’; if there is a linking function, who fulfils this role; the length or the pathway or support provided to CYP; and the type of activity offered to CYP as part of the pathway. Of the 68 sources included in this review, 72 separate social prescribing services were described (some sources included more than one organisation). We are aware that there are often multiple pathways through social prescribing after the point of entry, but sources did not report on this.

#### Referrers

There was a wide range of referral sources, the most common of which were educational institutions (*N* = 31), GPs (*N* = 29) and self-referral (*N* = 27). Others included referrals from mental health services, within-service (internal), parents, family, social services, friends and other healthcare professionals/services. Some services included multiple referral sources.

#### Linking function

An explicit Link Worker/social prescribing function was described for 25 services (i.e. a third), using various terms from simply ‘Link Worker’ to ‘Young Person’s Social Prescriber and Link Worker’.

Twenty services did not appear to have a specific linking function but met our inclusion criteria by providing access to community assets as a way of improving health and wellbeing. The linking function was unclear in the remaining six organisations.

The linking function was generally broad and described the role of the person working at the service who provided young people with access to community assets. Perhaps unsurprisingly given the common settings, this was mostly done by school staff, mentors and youth workers.

#### Length of pathway/support

There was little commonality in the length of support offered, either directly by the service or by the pathway from referrer to activity overall, with this varying widely from six weeks to twelve years where reported (*N* = 38 not reported). Several services reported the number of sessions offered but largely no timeframe over which these was provided.

#### Type of activity offered

Thirty-six services reported offering support to young people which fell outside of the formal ‘pillars’ described by NASP, with some not stating anything specific (e.g., ‘community engagement’) [3], and some describing activities that could have the potential to improve wellbeing, but which did not directly fall within the four pillars (e.g., youth clubs, peer support groups, volunteering, links to fashion and beauty) (No [38]. We subsequently split this ‘other’ category based on our reading of included papers and included the nuance between: (i) other social groups not captured above, and (ii) other support not captured above.

Services providing activities which fell within the four pillars were fairly evenly distributed across physical activity, advice and information, and arts and heritage, with some providing activities relating to more than one of these categories. Thirty services reported offering physical activity with 10 of the 11 services offering nature-based activities also explicitly including physical activity. Twenty-eight services offered links to advice-based support, and 24 services offered arts-based activities.

Physical activity included activities such as surfing, yoga, sports, and ‘walk and talk’. Bush therapy, gardening and ecotherapy were described as examples of natural environment. Advice and information included practical housing, family and educational/careers advice, as well as things like mentoring. Examples of arts and heritage included creative art, theatre and street dance.

## Discussion

We aimed to comprehensively map the existing evidence relating to how social prescribing services operate as a vehicle for assisting CYP with mental health difficulties to access, connect with and participate in community activities. Our broad scope ensured maximum coverage of what is an under-researched field. Included studies detailed a very broad range of approaches to delivering social prescribing, a diversity that in some ways matches and in other ways is distinct from the descriptions of adult services reported elsewhere [19]. In the sections below we outline this complexity and also the completeness and applicability of the evidence base more broadly.

### Diversity of approaches

The included evidence indicated that for CYP experiencing or at risk of mental health issues, social prescribing pathways are directed at myriad cohorts of individuals. In general, this range was focused on those at risk of or experiencing mental ill health ranging from low mood and loneliness through to complex severe mental health issues and trauma – however, a significant number were vague in their description of the presenting difficulty. Mental health is described and considered in amorphous ways, this is similar in adult social prescribing but has ramifications for how we think about eligibility, and the success of CYP SP (i.e., prevention, recovery) and routes of access [1]. This lack of specificity has implications for early intervention services and sectors seeking to respond to early risks for poor mental health [21].

The diversity reported in delivery across sectors and services in this review differed from adult-focused (and all-age offered) social prescribing programmes. Whilst there is certainly variability in all-age offers, most services focus on primary care, particularly general practice as the referring organisation, and a PCN or health-service employed link worker as providing the linking function. However, in the evidence reported in this review, we note that pathways are often broader and include school, self-referrals and community organisations as first points of contact for CYP; rather than mainly primary care.

Linked to this, there was diversity in the linking function itself, which was delivered through a breadth of role functions and forms, such as young person specific link workers, youth workers, or other community employed roles. There were quite a few schemes where there was no dedicated linking function at all. Where this is the case, the young person was usually only able to access support or activities that were provided by the organisation referred to at the outset. Therefore, this is not a genuinely needs-based approach to social prescribing. Given this, it is likely that a formal linking function, provided regardless of role, potentially adds a positive element of matching; tailoring and personalising services through assessments of needs, severity and fit – again something that reflects the broader social prescribing literature [45] and wider healthcare literature (e.g., [10, 13, 35].

In terms of the community activities that CYP engaged in, unlike all-age offers, the majority of reported community activities for CYP fell outside the four pillars most often reported, with most providing a more general offer and community engagement alongside social support. We would argue that this framework is not the best way of categorising community offers for CYP and some reframing would be useful in future research to capture this broader range of activities. The categories we created in response to the data extracted were other social groups not captured in the four pillars, and other support which was not captured in the four pillars. This included activities such as volunteering and mentoring. This likely is a reflection of the range of activities available for CYP in local spaces and directed by CYP preference; where activities in other categories such as arts and heritage or the natural environment may be less likely to be developed or designed for CYP and are perhaps catered to in other ways such as through school or specific interventions such as forest school.

### Completeness and applicability of the evidence base

Given this is a mapping review and not a review of effectiveness or impact, we reported the breadth of the evidence but did not conduct analyses of differential implementation or impact across geographies. Most included studies reported on programmes active in England, across a range of local and regional areas, with fewer but still multiple studies reporting on activity in Scotland and Wales. Internationally, there was evidence relating to multiple regions of Canada and Australia, the USA, Finland, Israel, Jamaica, and New Zealand. This reflects the growing move towards SP-like schemes reported in global analyses [28].

The overall volume of evidence located in this review (*N* = 68) is significantly more than recent reviews that have focused solely on programmes termed ‘social prescribing’ (e.g., [16, 17], and we feel this breadth gives context and important detail to these reviews. Similarly, and in keeping with other reviews on linked topics [8], a great deal of this evidence lay outside of the formal published academic literature in grey sources. Again, whilst this is a mapping rather than effectiveness review, we note the overall positive impacts on participants reported in included studies. This observation aligns with the findings of a recent scoping review which reported increases in mental, physical, and social health and reductions in healthcare demand across several evaluations of social prescribing for CYP [29].

### Implications for CYP-SP sector

This findings of this review point to several areas for consideration. Firstly, the need for clearer articulation around who social prescribing services serve, are implemented for, and directed to; while we were specifically interested in CYP at risk or experiencing mental health issues, this was described in a variety of ways by social prescribing services. Greater clarity around the mental health needs which may be best supported by interventions such as social prescribing will support access to these services and will enable those referring to services to utilise them more effectively to support prevention and recovery.

Second, our review included social prescribing services regardless of whether a linking function was described. We did this to enable us to capture a wider range of social prescribing activities and not only those which adhere to the model commonly seen in adult social prescribing where there is a clear linking function and role. There were several included services where a linking function was not present, unlike many adult social prescribing models. This is important to consider as we learn more about what social prescribing looks like and should look like for CYP, whether this is an important and helpful distinction or whether the absence of a linking function may be problematic in terms of achieving the aims of social prescribing for the individual. A lack of linking function may be limiting in terms of the community assets or activities they might consider and also disables features such as shared decision-making which are central to other social prescribing models and important to ensure youth-led and person-centred approaches [32]. Wider literature also supports the individualisation of approaches to healthcare and interventions for young people [10, 13]. The individual responsible for the linking may also be a limiting factor, while a link worker functions specifically to guide the young person to activities of interest, other individuals performing the linking function, for example, a nurse or a teacher, may have a specific agenda dictated by their work role, or may be less likely to have detailed knowledge of local services or activities, thereby potentially limiting what can be offered to the young person and/or how this linking can be facilitated i.e., processes such as shared decision making.

Finally, our review supports the need to consider frameworks such as the four pillars [31]. Our findings suggest that these four pillars do not adequately cater to the range of activities which feature in social prescribing for CYP. While understanding of adult social prescribing is far more established than the CYP literature, our understanding of what social prescribing looks like, or indeed, should look like in terms of pathways, and what social prescribing includes, in terms of community assets or activities for CYP warrants further exploration.

### Strengths and limitations

The key strength of this systematic mapping review lies in its comprehensive scope and inclusive methodology. By casting a wide net across both academic and grey literature, the review captures a rich and diverse set of approaches to social prescribing for CYP with mental health needs. Within this, the terms used to explore social prescribing for CYP did not focus solely on those named as ‘social prescribing’. While this would have provided a narrower focus, many social prescribing activities or programmes for CYP do not use this terminology. Using more inclusive terms provides additional context and detail to the picture of social prescribing activities in this space. This breadth enables a nuanced understanding of the variety in referral routes, linking roles, and types of community activities, offering valuable insights into how SP is currently implemented and conceptualised for CYP.

The review highlights inconsistencies in how mental health needs are defined and addressed, raising critical questions about eligibility, access, and tailoring of services. However, this wide scope also presents limitations. As a mapping review, it does not evaluate effectiveness or differential outcomes across contexts, which constrains its ability to inform evidence-based practice or policy. Additionally, the inclusion of programmes without a defined linking function may dilute the conceptual clarity of SP and limit interpretability around what constitutes effective practice. The lack of a cohesive framework for categorising CYP-specific community activities—such as the inadequacy of the “four pillars” model—further reflects the nascent state of the evidence base and highlights the need for more tailored evaluative frameworks. Overall, while the review provides a strong foundation for understanding the diversity and potential of CYP-SP, it also underscores the need for clearer definitions, more rigorous evaluations, and CYP-specific conceptual models.

## Conclusion

This is the only review to map the ways in which social prescribing schemes operate as a vehicle for assisting CYP with mental health difficulties to access, connect with and participate in community activities. Included studies (*N* = 68) reported important distinctions from adult or all-age offered social prescribing services: a broader range of referral organisations, and link worker functions across more sectors and organisations. There was also a difference in the type of community activities CYP engaged in, with more individuals participating in less focused/specialised activities centred on nature, or arts, and more generalised social or support groups. Future research should examine how and in what ways particular cohorts (including those often under-served) access, or not, these sorts of pathways, the economic impact, and examine any potential risks or harms that might be associated with CYP-SP.

## Supplementary Information

Supplementary Material 1.

Supplementary Material 2.

Supplementary Material 3.

Supplementary Material 4.

Supplementary Material 5.

## Data Availability

No datasets were generated or analysed during the current study.
