# Supplementary material for: The use of social prescribing and community-based wellbeing activities as a potential prevention and early intervention pathway to improve adolescent emotional and social development: a systematic mapping review
Source: BMC Public Health. 2025 Oct 15;25:3495. doi: 10.1186/s12889-025-24413-5 (PMC12522731; doi:10.1186/s12889-025-24413-5)
Supplement: Supplementary file 1 — Supplementary Material 1. [file 12889_2025_24413_MOESM1_ESM.docx]

*Medline Search Strategy*

1 Child/ (1817133)
2 Adolescent/ (2158796)
3 Young Adult/ (979277)
4 Students/ (70372)
5 youth*.tw. (86004)
6 teen*.tw. (32851)
7 (young* adj2 (adult* or person* or individual* or people* or population* or man or mans or men or mens or woman* or women* or male or males or female*)).tw. (282945)
8 youngster*.tw. (2640)
9 (child or children*).tw. (1347600)
10 (boy or boys).tw. (155906)
11 girl*.tw. (161397)
12 student*.tw. (320480)
13 pupil*.tw. (31753)
14 school*.tw. (313275)
15 adolescen*.tw. (313506)
16 1 or 2 or 3 or 4 or 5 or 6 or 7 or 8 or 9 or 10 or 11 or 12 or 13 or 14 or 15 (4476988)
17 Mental Health/ (50731)
18 *Anxiety/ (47283)
19 *Depression/ (82940)
20 *Stress, Psychological/ (80529)
21 *Psychological Distress/ (2181)
22 (mental adj2 health).tw. (177861)
23 (mental adj ill*).tw. (36496)
24 (mental adj well*).tw. (4836)
25 (mental adj2 stress*).tw. (5813)
26 (mental adj2 distress).tw. (2913)
27 (psycholog* adj2 health).tw. (11091)
28 (psycholog* adj ill*).tw. (562)
29 (psycholog* adj well*).tw. (13020)
30 (psycholog* adj2 stress*).tw. (11843)
31 (psycholog* adj2 distress*).tw. (24442)
32 depress*.tw. (509644)
33 anxious*.tw. (19162)
34 anxiet*.tw. (226349)
35 camhs.tw. (558)
36 17 or 18 or 19 or 20 or 21 or 22 or 23 or 24 or 25 or 26 or 27 or 28 or 29 or 30 or 31 or 32 or
3 or 34 or 35 (885166)
37 16 and 36 (256408)
38 (natur* or art or arts or drama* or theatre*).ti. (209747)
39 (Therap* or program* or coordinat* or intervention or referral* or prescrib* or prescrip* or facilit* or connect* or coordinat* or activ* or treatment*).tw. (12318357)
40 38 and 39 (83570)
41 (natur* or art or arts or drama* or theatre*).ab. (1443355)
42 (Therap* or program* or coordinat* or intervention or referral* or prescrib* or prescrip* or facilit* or connect* or coordinat* or activ* or treatment*).ab. (11064374)
43 ((natur* or art or arts or drama* or theatre*) adj2 (Therap* or program* or coordinat* or intervention or referral* or prescrib* or prescrip* or facilit* or connect* or coordinat* or activ* or treatment*)).ab. (44051)
44 (forest* or adventur* or wilderness* or wellbeing or well being or outdoor* or sport* or walk* or danc* or yoga or tai chi or pilates or game or gaming or relaxation or breathing or entertainment* or reading or book* or music* or religi* or mindfulness or spiritual or meditation or retreat* or reflexology or reiki or massage or volunteer* or hobby or hobbies or pet or pets or creativ* or draw* or paint* or garden* or farm* or horticultur* or complementary or soccer or football or writing or journal* or exercis* or housing or financ* or weight or computer or virtual reality).tw. (3263818)
45 ((forest* or adventur* or wilderness* or wellbeing or well being or outdoor* or sport* or walk* or danc* or yoga or tai chi or pilates or game or gaming or relaxation or breathing or entertainment* or reading or book* or music* or religi* or mindfulness or spiritual or meditation or retreat* or reflexology or reiki or massage or volunteer* or hobby or hobbies or pet or pets or creativ* or draw* or paint* or garden* or farm* or horticultur* or complementary or soccer or football or writing or journal* or exercis* or housing or financ* or weight or computer or virtual reality) adj2 (Therap* or program* or coordinat* or intervention or referral* or prescrib* or prescrip* or facilit* or connect* or coordinat* or activ* or treatment*)).tw. (155195)
46 (physical activ* adj2 (Therap* or program* or coordinat* or intervention or referral* or prescrib* or prescrip* or facilit* or connect* or coordinat* or treatment*)).tw. (7388)
47 40 or 43 or 45 or 46 (280831)
48 47 and 37 (6173)
49 (social* adj2 referral*).tw. (270)
50 (communit* adj2 referr*).tw. (1284)
51 (communit* adj2 prescrip*).tw. (237)
52 (communit* adj2 prescrib*).tw. (361)
53 (social* adj2 prescrib*).tw. (484)
54 (social* adj2 prescrip*).tw. (91)
55 (Care adj2 navigator*).tw. (114)
56 (Wellbeing adj2 coordinator*).tw. (3)
57 (Referral adj2 facilitator*).tw. (28)
58 (Community adj2 connector*).tw. (17)
59 49 or 50 or 51 or 52 or 53 or 54 or 55 or 56 or 57 or 58 (2820)
60 (pathway* or barrier* or facilitat* or service* or provider* or connector* or coordinator* or navigator* or communit* or social* or volunteer*).tw. (3889300)
61 48 and 60 (2751)
62 (pathway* or barrier* or facilitator*).ti. (305988)
63 (pathway* or barrier* or facilitator*).ab. (1533417)
64 (service* or provider*).tw. (725353)
65 (service* or provider*).ab. (640823)
66 62 and 64 (10558)
67 ((pathway* or barrier* or facilitator*) adj2 (service* or provider*)).ab. (2408)
68 66 or 67 (12353)
69 37 and 68 (862)
70 37 and 59 (235)
71 61 or 69 or 70 (3829)
