# Supplementary material for: The use of social prescribing and community-based wellbeing activities as a potential prevention and early intervention pathway to improve adolescent emotional and social development: a systematic mapping review
Source: BMC Public Health. 2025 Oct 15;25:3495. doi: 10.1186/s12889-025-24413-5 (PMC12522731; doi:10.1186/s12889-025-24413-5)
Supplement: Supplementary file 2 — Supplementary Material 2. [file 12889_2025_24413_MOESM2_ESM.docx]

Call for evidence websites

<https://www.greenteam.org.uk/our-programmes/thrive/>

<https://www.resurgamtrust.co.uk/health-d1/>

<https://brutonsurgery.nhs.uk/>

[www.onside-advocacy.org.uk](http://www.onside-advocacy.org.uk)

<https://www.youngdevon.org/>

<https://www.sheffieldfutures.org.uk/>

<https://www.tidenhamparishchurch.co.uk/whats-going-on/youth-children-and-families/>

Grey Literature search websites

| **Website/service name** | **URL** |
| --- | --- |
| **Anna Freud Centre** | <https://www.annafreud.org/> |
| **Street Games** | <https://www.streetgames.org/> |
| Family Engagement Project | <https://www.streetgames.org/family-engagement-project> |
| London Safer Together Through Sport | <https://www.streetgames.org/london-safer-together-through-sport> |
| Sport for Better Mental Health | <https://www.streetgames.org/brunel-university-sport-for-better-mental-health> |
| Fit and Fed | <https://www.streetgames.org/fit-and-fed-summer-2018-report> |
| Doorstep Sport Club | <https://www.streetgames.org/insight-into-action> |
| Us Girls Wales Learning | <https://www.streetgames.org/us-girls-wales-learning> |
| **Social Prescribing Network** | <https://www.socialprescribingnetwork.com/> |
| Polley, M et al. Evaluation of the Reading Well for Young People Scheme. 2017 | <https://westminsterresearch.westminster.ac.uk/download/dca7ff61099254ce1469cdcb76e92f65e6d62ab906fb0c73a5d8c8dcefe6096f/1435429/Reading%20Well%20for%20young%20people%20evaluation.pdf> |
| Bertotti , M. et al. A two-year evaluation of the Young People Social Prescribing (YPSP) pilot An outcome, process and economic evaluation of social prescribing for young people in three English sites, Institute for Connected Communities (ICC), University of East London, December 2020 | <https://repository.uel.ac.uk/download/5c63906437d90e4093a320b51355232d12648ab00d6df93137b1aefa74dfc892/851983/SP%20for%20young%20people%20evaluation%20final%20report%20for%20publication.pdf> |
| **National Association of Social Prescribing** | <https://socialprescribingacademy.org.uk/> |
|  | <https://socialprescribingacademy.org.uk/internationalyouthday/> |
|  |  |
|  |  |
| **Youth Endowment Fund** | https://youthendowmentfund.org.uk/ |
|  |  |
| **Nesta** | <https://www.nesta.org.uk/> |
| **Early Intervention Foundation** | <https://www.eif.org.uk/> |
| **The Kings Fund** | <https://www.kingsfund.org.uk/> |
| **Barnardos** | <https://www.barnardos.org.uk/> |
| LINK Cumbria | <https://www.barnardos.org.uk/what-we-do/services/cumbria-link-young-peoples-social-prescribing-service> |
| **Save the Children** | <https://www.savethechildren.org.uk/> |
| **NSPCC** | <https://www.nspcc.org.uk/> |
| **National Lottery Community Fund** | <https://www.tnlcommunityfund.org.uk/insights/social-prescribing> |
| **Association for Young People’s Health (AYPH)** | <https://www.youngpeopleshealth.org.uk/> |
| **Mind** | <https://www.mind.org.uk/news-campaigns/campaigns/children-and-young-peoples-mental-health/> |
| **UK Youth.org** | <https://www.ukyouth.org/> |
| **Faith Action** | <https://www.faithaction.net/> |
| **Health Foundation** | <https://www.health.org.uk/> |
| **Sport England** | <https://www.sportengland.org/> |
| **London Sport** | <https://londonsport.org/> |
| **NCVO** | <https://www.ncvo.org.uk/> |
| **Work Foundation** | <https://www.lancaster.ac.uk/work-foundation/> |
| **Joseph Rowntree Foundation** | <https://www.jrf.org.uk/> |
| **Wellcome Trust** | <https://wellcome.org/> |
| **WHO** | <https://www.who.int/> |
| **Mental Health Foundation** | <https://www.mentalhealth.org.uk/> |
| **Institute for Volunteering Research** | <https://www.uea.ac.uk/groups-and-centres/institute-for-volunteering-research> |
| **British Library social welfare portal** | <https://www.bl.uk/social-welfare> |
| **SCIE (Social Care Institute for Excellent) - Social care online** | <https://www.scie-socialcareonline.org.uk/> |
| **NHS England** | <https://www.england.nhs.uk/> |
| **Young Minds** | <https://www.youngminds.org.uk/> |
| **Green Team (Thrive)** | [https://www.greenteam.org.uk/our-programmes/thrive/](https://eur03.safelinks.protection.outlook.com/?url=https%3A%2F%2Fwww.greenteam.org.uk%2Four-programmes%2Fthrive%2F&data=04%7C01%7Calex.gude%40plymouth.ac.uk%7C7cb363309ecd4a72b32708d9b8942b1e%7C5437e7eb83fb4d1abfd3bb247e061bf1%7C1%7C0%7C637743769239127716%7CUnknown%7CTWFpbGZsb3d8eyJWIjoiMC4wLjAwMDAiLCJQIjoiV2luMzIiLCJBTiI6Ik1haWwiLCJXVCI6Mn0%3D%7C2000&sdata=9Wl%2FNOA%2F6XSguGonH%2FneylUL4ZOMYWimWTeD%2BPpPONk%3D&reserved=0) |
| Green Team (Green Volunteers) | <https://www.greenteam.org.uk/our-programmes/green-volunteers/> |
| Green Team (Green Angels) | <https://www.greenteam.org.uk/our-programmes/green-angels/> |
| Green Team (Green Explorers) | <https://www.greenteam.org.uk/our-programmes/green-explorers/> |
| Green Team (Green Shoots) | <https://www.greenteam.org.uk/our-programmes/green-shoots/> |
| Green Team (Green Schools) | <https://www.greenteam.org.uk/our-programmes/green-schools/> |
| Green Team (Nature Play) | <https://www.greenteam.org.uk/our-programmes/nature-play/> |
| Green Team (Holiday Activity) | <https://www.greenteam.org.uk/our-programmes/holiday-activity/> |
| Green Team (Green Champions) | <https://www.greenteam.org.uk/our-programmes/green-champions/> |
| Green Team (Green Wellies) | <https://www.greenteam.org.uk/our-programmes/green-wellies/> |
| Green Team (Green Connections) | <https://www.greenteam.org.uk/our-programmes/green-connections/> |
| **Tidenham Parish Council - Community Youth Engagement Specialist** | https://www.tidenhamparishcouncil.co.uk/ |
| **Young Devon** | https://www.youngdevon.org/news/community-connectors |
| **Resurgam Healthy Living Centre** | <https://www.resurgamtrust.co.uk/health-d1/> |
| **Bruton GP Practice, Somerset . Bruton Health Coach plan.** | <https://brutonsurgery.nhs.uk/health-coaches/> |
| **Community Development Manager** | [www.onside-advocacy.org.uk](http://www.onside-advocacy.org.uk/) |
| **Well London** | [Well London - Home](http://www.welllondon.org.uk/) |
| **Uthink project** | [(PDF) Evaluation of the UThink Project 1 Promoting Positive Mental Health and Recovery in Young People: Evaluation of the UThink Project FINAL REPORT FOR RETHINK \| Nigel Thomas - Academia.edu](https://www.academia.edu/3139342/Evaluation_of_the_UThink_Project_1_Promoting_Positive_Mental_Health_and_Recovery_in_Young_People_Evaluation_of_the_UThink_Project_FINAL_REPORT_FOR_RETHINK) |
| **Formerly YES (The Zone)** | <https://www.thezoneplymouth.co.uk/> |
| **Holistic Arts-Based Program (HAP)** | <https://dianacoholic.com/> |
| **Student Minds** | <https://www.studentminds.org.uk/whatwedo.html> |
| **Jigsaw** | See papers saved in grey lit folder |
| **Sistema Scotland’s“Big Noise”orchestral programme** | <https://makeabignoise.org.uk/> |
| **Hope and Horizon Woodland Retreat** | <https://www.blueforest.com/made-to-stay/hope-and-horizon-woodland-retreat/> |
| **Jamie’s Farm** | <https://jamiesfarm.org.uk/> |
| **Sing up** | <https://www.singup.org/about-us> |
| **Navigate your health** | <https://www.childrens.health.qld.gov.au/service-navigate-your-health/> |
| **Parkrx** | <https://www.parkrx.org/> |
| **YN/RA** | <http://ynra.ca/> |
| **YMCA Exeter** | https://www.ymcaexeter.org.uk/i-need-support/resilience/community-connecting/ |
| **Connected communities** | <https://connected-communities.org/index.php/about/#:~:text=Connected%20Communities%20was%20a%20multi,ran%20from%202009%20to%202019.> |
| **Police citizens youth clubs** | <https://www.pcycnsw.org.au/programs/rise-up> |
| **FAST** | <https://www.familiesandschools.org/> |
| **1625 independent people** | <https://www.1625ip.co.uk/> |
| **My Strengths Training for Life (MST4Life)** | <https://www.sprintproject.org/projects> |
| **Wave by wave surf therapy** | <https://gulbenkian.pt/en/news/wave-by-wave-surf-as-therapy/> |
| **Youth Skateboarding Program** | <http://indigoyouthmovement.org/> |
| **Thriving Not Just Surviving** | <https://www.centreformentalhealth.org.uk/news/creative-and-sporting-activities-make-mental-health-support-more-accessible-boys-and-young-men-says-centre-mental-health-research> |
| **MYPAS (Midlothian Young People's Advice Service)** | https://www.mypas.co.uk/young-mens-mental-health-project/ |
| **LifeSet (via Youth Villages)** | <https://youthvillages.org/services/lifeset/> |
| **Project ASSERT Boston Medical Center** | https://www.bmc.org/project-assert |
| **Nature Nurture Aberdeen** | https://salugen.uk/?fbclid=IwAR1IQ8oF8ZISVmKQv9Y2ht5GTsH7bgFaXCtB0OrQSopWhRYKN6_Sn6_nXNw |
| **Aberdeen Foyer** | https://www.aberdeenfoyer.com/ |
| **Building Bridges Newcastle** | None |
| **Club Deportivo Dan (Voices4Peru)** | https://v4peru.org/?fbclid=IwAR0yKjPIDzJvpzGdLwS1DOxs3RvBrIOEHIrwyAx4_Eq9I1ccO1Q-1cKb5uI |
| **Grassroot Gardens of Western New York** | https://www.grassrootsgardens.org/ |
| **Frames Film Project Vancouver** | https://www.froghollow.bc.ca/programs-services/youth/frames-film-project/ |
| **BAM! Boys Advocacy and Mentoring Programme** | https://bamgroups.com/index.html |
| **Association for Young People's Health** | [https://ayph.org.uk](https://ayph.org.uk/) |
| **Children & Young People's Mental Health Coalition** | [https://cypmhc.org.uk](https://cypmhc.org.uk/) |
| **Jigsaw - Irish youth suicide prevention service (identified while screening full text paper 'Connecting for Life'** | |
| **what works wellbeing centres** | https://whatworkswellbeing.org/ |
| **NHS Futures SP Platform** |  |
| Linking Leeds | Evaluation document on NHS Futures |
| Macmillan social prescribing | Project info document on NHS Futures |
| Green Social Prescribing - Mindful Ninja | Project info document on NHS Futures |
| Kensington and Chelsea Social Council (KCSC) Self-care SP | Project info document on NHS Futures |
| **Reaching Adolescents for Prevention - The Role of Pediatric Emergency Department Health Promotion Advocates** | Reaching Adolescents for Prevention: The Role of Pediatric E... : Pediatric Emergency Care (lww.com) https://www.reliasmedia.com/articles/141101-health-promotion-advocate-extends-benefits-offered-to-adolescents-young-adults |
| **Headspace** | https://headspace.org.au/ |
| **Orygen** | <https://www.orygen.org.au/About/About-Us> |
| **Take Art** | <https://takeart.org/> |
| **Outthere** | Project page via Take Art |
| **Making & Believing** | Project page via Take Art |
| **Little Big Dance** | Project page via Take Art |
| **The SoundWaves Network** | Project page via Take Art |
| **My Tunes** | Project page via Take Art |
| **Love media love arts programme** | <https://loveorganization.ca/qc/en/programs> |
| **Various other services mentioned in Listen Up report (lines 114 - 122)** | <https://www.bl.uk/britishlibrary/~/media/bl/global/social-welfare/pdfs/non-secure/l/i/s/listen-up-personcentred-approaches-to-help-young-peopl-experiencing-mental-health-and-emotional-problems.pdf> |
| **Brighter Futures, Reading** | <https://brighterfuturesforchildren.org/?s=link+worker&post_type=page> |
| **The Junction, Colchester** | [https://thejunctionfoundation.com/services/#](https://thejunctionfoundation.com/services/) |
| **Community Links, Canning Town** | <https://www.community-links.org/youth-employment/> |
| **Aberdeen Foyer** | <https://www.aberdeenfoyer.com/services/housing-for-young-people/> |
| **Caterpillar Service, Barnardo's Cardiff** |  |
| **Streetwise, Newcastle** | <https://www.streetwisenorth.org.uk/> |
| **Icebreak, The Zone, Plymouth** | <https://www.thezoneplymouth.co.uk/mental-health-and-wellbeing/icebreak> |
| **The Market Place, Leeds** | <https://www.themarketplaceleeds.org.uk/> |
| **Experience in Mind, Mind Brighton & Hove YMCA** |  |
| **Mind the Gap Brighton & Hove** | <https://www.brightonandhovewellbeing.org/cyp-mind-the-gap> |
| **YMCA Downslink** | <https://www.ymcadlg.org/what-we-do/support-and-advice/yac-housing-advice-for-young-people/> |
| **Senior Research Fellow - Co-production and Citizen Science , Honorary RF University of Bradford** | Islam 2011 Square pegs in round holes: the mental health needs of young adults and how well these are met by services - an explorative study |
| **Senior Research Fellow - Co-production and Citizen Science , Honorary RF University of Bradford** | Islam et al 2019 Assessing community readiness for early intervention programmes to promote social and emotional health in children https://doi.org/10.1111/hex.12887 |
| **Nordoff Robbins** | <https://www.nordoff-robbins.org.uk/about-us/public-policy/social-prescribing/> |
| **No Limits Southampton** | <https://nolimitshelp.org.uk/get-help/health-wellbeing/social-prescribing/> |
| **Hartlepower (Hartlepool)** | <https://hartlepower.co.uk/youth-social-prescribing> |
| **Family Action Hackney London** | <https://www.family-action.org.uk/what-we-do/children-families/emotional-health-wellbeing-services__trashed/> |
| **Live Well Greater Manchester** | ? |
| **Active Luton - Life Hacks** | <https://www.activeluton.co.uk/young-persons-social-prescription> |
| **Tower Hamlets** | <https://www.towerhamletstogether.com/our-work/social-prescribing-service> |
| **Here4YOUth Dudley** | <https://cranstoun.org/help-and-advice/young-people/here4youth-dudley/> |
| **Youth Clinic Fellowship Project Battersea London** | ? |
| **Battersea Youth Clinic** | <https://www.battersearisegrouppractice.co.uk/navigator/book-an-appointment-with-our-youth-link-worker/> |
| **Zone West Newcastle** | <https://www.northeastwellbeing.co.uk/zone-west/> |
| **Rochdale FE College** | ? |
| **ActOnIt via Onside Advocacy Worcester** | <https://www.onside-advocacy.org.uk/actonit> |
| **KAOS London** | <https://www.redthread.org.uk/what25meanstome-kaos/> |
| **Salford College** | <https://gmbeewell.org/beewell-champions-social-prescribing-pilot-to-launch-in-autumn-2022/> |
| **Isledon Arts CIC - Lift** | <https://www.liftislington.org.uk/events/social-prescribing-team> |
| **Isledon Arts CIC - Rose Bowl** | <https://www.rosebowlislington.org.uk/> |
| **Well Centre Lambeth** | <https://www.thewellcentre.org/what-to-expect/> |
|  | https://www.thewellcentre.org/referrals/ |
| **Three Rivers Academy** | <https://www.threeriversacademy.org/Information/Home-School-Link-Worker/> |
| **Kinross High School** | <https://www.kinrosshighschool.org.uk/parents/advice-and-resources-for-parentscarers/community-link-worker/> |
| **Street Games** | https://network.streetgames.org/our-work-changing-lives-health/youth-social-prescribing |
| **Headstart** | <https://www.ncb.org.uk/sites/default/files/uploads/attachments/Headstart%20report%20social%20prescribing_0.pdf> |
| **Manor mead Shepperton** | <https://www.manor-mead.surrey.sch.uk/Parents/Home-School-Link-Worker/> |
| **Sheffield Futures** | https://portervalleyprimarycarenetwork.gpweb.org.uk/index.php/pcn-services/item/young-person-s-social-prescribing-link-worker-door-43 |
| **St Mary's primary school** | <https://www.stmarys-byfleet.surrey.sch.uk/page/?title=Home+School+Link+Worker&pid=104> |
| **St Stephen's primary school** | <https://www.ststephens.surrey.sch.uk/parents/home-school-link-worker> |
| **Culm Valley** | https://www.culmvalleyyouthforum.co.uk/youth-link-worker |
| **Waverly Abbey Junior School** | <https://www.waverley-abbey.surrey.sch.uk/page/?title=Home+School+Link+Worker&pid=116> |
| **Stort Valley Healthcare Young Peoples Social Prescription Service** | <https://www.stortvalleyhealthcare.com/services/adolescent-health/> |
| **Buckinghamshire Council** | https://schoolsweb.buckscc.gov.uk/family-support-service/school-link-workers/ |
| **Claremont Bristol** | <https://www.claremontbristol.org.uk/Family-Support/> |
| **Community link worker project, Highland** | https://www.supportinmindscotland.org.uk/community-link-worker-project |
| **Saltergate schools** | <https://www.saltergateschools.co.uk/family-link-worker/> |
| **Dorset Youth** | https://www.dorsetyouth.com/Pages/FAQs/Category/staff |
| **Active Luton** | https://www.activeluton.co.uk/young-persons-social-prescription |
| **Healthy London** | <https://www.healthylondon.org/wp-content/uploads/2019/03/HLP-CYP-Social-Prescribing-for-CYP-Dec-2016.pdf> |
| **Imago** | <https://www.imago.community/Adult-Support/Social-Prescribing> |
| **Cumbria Link (Barnardos)** | https://www.barnardos.org.uk/what-we-do/services/cumbria-link-young-peoples-social-prescribing-service |
| **Headstart** | <https://www.ncb.org.uk/sites/default/files/uploads/attachments/Headstart%20report%20social%20prescribing_0.pdf> |
| **Stort Valley Healthcare Young Peoples Social Prescription Service** | <https://www.stortvalleyhealthcare.com/services/adolescent-health/> |
| **Social prescribing in children and young people - UCL** | <https://www.ucl.ac.uk/evidence-based-practice-unit/sites/evidence-based-practice-unit/files/review_social_prescribing_in_children_and_young_people_final_0.pdf> |
| **Anna Freud Centre** | <https://www.annafreud.org/on-my-mind/get-involved/take-part-in-research/previous-projects-and-research/completed-understanding-social-prescribing-for-children-and-young-people-to-improve-mental-health-and-wellbeing/> |
| **The Guardian** | <https://www.theguardian.com/society/2022/oct/25/young-people-offered-surfing-dancing-nhs-help-anxiety> |
| **Barts Charity** | <https://www.bartscharity.org.uk/our_news/piloting-a-model-of-social-prescribing-for-young-people/> |
| **Healthy London** | <https://www.healthylondon.org/wp-content/uploads/2019/03/HLP-CYP-Social-Prescribing-for-CYP-Dec-2016.pdf> |
| **Emerging Minds** | <https://emergingminds.org.uk/developing-social-prescribing-to-improve-mental-health-and-wellbeing-for-children-and-young-people/> |
| **The Acorn Practice** | <https://theacorngrouppractice.co.uk/social-prescribing/> |
| **Linking Leeds** | <https://www.ncbi.nlm.nih.gov/pmc/articles/PMC8835307/> |
| **StreetGames** | <https://network.streetgames.org/resources/young-peoples-social-prescribing-resources> |
| **The Joy App** | <https://www.thejoyapp.com/articles/social-prescribing-its-not-just-for-the-older-generation> |
| **Stort Valley Healthcare** | <https://www.stortvalleyhealthcare.com/services/adolescent-health/> |
| **Living Well UK** | <https://livingwellconsortium.com/services/community-wellbeing/> |
| **Barnardo's** | <https://www.barnardos.org.uk/commission-us/social-prescribing> |
| **IMAGO** | <https://www.imago.community/Adult-Support/Social-Prescribing> |
| **Groundwork** | <https://www.groundwork.org.uk/green-social-prescribing/green-social-prescribing-supporting-people-with-mental-ill-health/> |
| **UEL Repository (Bertotti 2 year evaluation of YP SP)** | <https://repository.uel.ac.uk/item/88x15> |
| **NHS England SP Pathway** | <https://mcusercontent.com/ec5dea9536bde16d5a3153530/files/ce706604-1578-1ca4-9b6a-2df5575c9f21/VR_SP_pathway_Paper_FINAL_FOR_PUBLICATION.pdf> |
| **Centre for Welfare** | <https://citizen-network.org/uploads/attachment/339/social-prescribing-for-mental-health.pdf> |
| **One Ellesmere Port PCN** | <https://oneellesmereport.co.uk/social-prescribing/> |
| **BHF** | <https://www.bhf.org.uk/informationsupport/heart-matters-magazine/wellbeing/social-prescribing> |
| **Kingswood Health Centre** | <https://www.kingswoodhealthcentre.co.uk/services/social-prescribing/> |
| **Safety Nets - Yorkshire Sport** | <https://www.yorkshiresport.org/what-we-do/children-young-people/safety-nets/> |
| **Wales Youth SP** | <https://static1.squarespace.com/static/5f020c49b484e47001f2bb5b/t/60ba5655cefb6743335f1d4a/1622824535346/walesyouthsocialprescribingarapidreview_2021.pdf> |
| **Gloucestershire CCG** | <https://www.gloucestershireccg.nhs.uk/nhs-and-partners-trial-an-innovative-preventative-programme-utilising-one-of-the-first-childrens-social-prescribing-schemes/> |
| **New Forest PCN** | <https://newforestpcn.co.uk/network-services/socialprescribing/youth> |
| **Brightstar** | <https://www.brightstarboxing.co.uk/blog/what-is-social-prescribing/> |
| **Scottish Government** | <https://www.gov.scot/publications/resources-mental-health-wellbeing-primary-care-services/pages/6/> |
| **No Limits** | <https://nolimitshelp.org.uk/get-help/nhs-partnerships/> |
| **YP's SP North Cotswolds** | <https://www.glosfamiliesdirectory.org.uk/kb5/gloucs/glosfamilies/service.page?id=4I13l7hvGqI&familychannel=1_1> |
| **Brandon Centre** | <https://brandon-centre.org.uk/services/social-prescribing> |
| **Yorkshire youth & music** | <https://www.yym.org.uk/social-prescribing> |
| **Chilypep** | <https://chilypep.org.uk/wp-content/uploads/2021/05/Open-Up-Barnsley-Directory-of-Services-2020.pdf> |
| **My Health** | <https://myhealthgroup.co.uk/your-care/managing-your-health/lifestyle-changes/social-prescribing/> |
| **SOFEA** | <https://www.sofea.uk.com/services/> |
| **Southmead.org** | <https://southmead.org/news/were-hiring-children-and-young-peoples-social-prescribing-link-worker> |
| **Southmead.org (Link Forward)** | <https://southmead.org/wellbeing/link-forward> |
| **COMIC** | <https://www.pcmis.com/news/article/comic-research-blog/> |
| **Mind Havant and East Hants** | <https://www.easthantsmind.org/wellbeing-services/children-and-young-peoples-wellbeing/primary-care-network-and-cyp-wellbeing-service/> |
| **Berkshire CCG** | <https://www.berkshirewestccg.nhs.uk/patient-information/self-care/social-prescribing/> |
| **Canford Health Group Practice** | <https://www.chgp.co.uk/info.aspx?p=4> |
| **Hall Street Medical Centre** | <https://www.hallstreetmedicalcentre.nhs.uk/digitalpractice/wellbeing-centre/mental-health/> |
| **Zoneworks (Zone West)** | <https://www.healthworksnewcastle.org.uk/service/zone-west/> |
| **Pentreath** | <https://www.pentreath.co.uk/projects/adults/social-prescribing/> |
| **Kilmorie Primary School** | <https://www.kilmorieschool.co.uk/parents/wellbeing-and-family-support/> |
| **Youth in Mind in City** | <https://youth-in-mind.org/youth-in-mind-in-city.php> |
| **HALE YP SP Project** | <https://haleproject.org.uk/reducing-isolation/young-peoples-social-prescribing-service/> |
| **Park Road Medical Practice** | <https://parkroadpracticewallsend.nhs.uk/managing-your-health/lifestyle-changes/social-prescribing/> |
| **Shropshire.gov** | <https://shropshire.gov.uk/committee-services/documents/s20228/9%20Social%20Prescribing.pdf> |
| **Jurassic Coast PCN** | <https://www.jurassiccoastpcn.nhs.uk/social-prescribing> |
| **Primary Care Training Hub** | <https://www.primarycaretraininghub.co.uk/socialprescribing> |
| **Active Essex** | <https://www.activeessex.org/health-wellbeing/social-prescribing/> |
| **West Yorkshire Partnership** | <https://www.wypartnership.co.uk/our-priorities/population-health-management/climate-change/green-social-prescribing-projects> |
| **Thrive** (different to Green Team above) | <https://rethinkingmedicine.org.uk/wp-content/uploads/2020/05/Read-more-about-Thrive.pdf> |
| **Linking Leeds** | <https://www.proquest.com/openview/14d31d89a25e36a658e48d67fc194698/1?pq-origsite=gscholar&cbl=54923> |
| **Three Spires Medical Practice** | <https://threespiresmedical.co.uk/info.aspx?p=12> |
| **Thriving Communities** | <https://www.communities1st.org.uk/what-thriving-communities> |
| **BeeWell** | <https://www.manchester.ac.uk/discover/news/youth-led-beewell-uncovers-major-insights-into-young-peoples-experiences-across-greater-manchester/> |
| **Youth Link** | <https://www.watfordfccsetrust.com/app/uploads/2021/06/Youth-Link-Referral-Criteria-Information-Sheet.pdf> |
| **Youth Link** | <https://www.watfordfccsetrust.com/project/youth-link/> |
| **Healthy Surrey** | <https://www.healthysurrey.org.uk/community-health/social-prescribing/green-social-prescribing> |
| **Wellbeing Exeter** | <https://www.stthomasmedicalgroup.co.uk/social-prescribing-for-young-people-11-17-through-wellbeing-exeter/> |
| **Bensham Family Practice** | <https://www.benshamfamilypractice.nhs.uk/help-and-support-service-types/mental-health/> |
| **Be Well Gloucester** | <https://www.bewellglos.org.uk/category/children-young-people-and-families/> |
| **Enable LC** | <https://enablelc.org/enable-lc-win-prestigious-social-prescribing-award/> |
| **Healthwatch Islington** | <https://www.healthwatchislington.co.uk/news/2019-09-30/improved-social-emotional-and-mental-health-offer-children-and-young-people> |
| **Gloucestershire Wildlife Trust** | <https://www.gloucestershirewildlifetrust.co.uk/blog/lorna-fox-head-community-programmes/green-social-prescribing> |
| **Cardiff University** | <https://www.cardiff.ac.uk/community/our-local-community-projects/case-studies/green-social-prescribing-in-the-cynon-valley> |
