# Supplementary material for: The use of social prescribing and community-based wellbeing activities as a potential prevention and early intervention pathway to improve adolescent emotional and social development: a systematic mapping review
Source: BMC Public Health. 2025 Oct 15;25:3495. doi: 10.1186/s12889-025-24413-5 (PMC12522731; doi:10.1186/s12889-025-24413-5)
Supplement: Supplementary file 3 — Supplementary Material 3. [file 12889_2025_24413_MOESM3_ESM.docx]

| **Author/year**  **Table 2. Synthesis of descriptive data about services from included papers** | **Service** | **Setting for delivery** | **Location** | **Age and other eligibility criteria** | **Reason for referral** |
| --- | --- | --- | --- | --- | --- |
| Appelqvist-Schmidlechner et al (2021) | Icehearts | At school, after school, at home | Finland (across 14 municipalities) | 6 – 18 years | Risk of social marginalisation |
| Bertotti et al (2020) | Young People Social Prescribing (YPSP) pilot | Various sites including wellbeing café | England (Sheffield) | 11 – 24 years | Emotional problems |
|  | Young People Social Prescribing (YPSP) pilot | YMCA, community centres, home visits (outreach) | England (Brighton & Hove) | 11 – 21 years | Social isolation |
|  | Young People Social Prescribing (YPSP) pilot | Home outreach, school | England (Luton) | 11 – 18 years | Anxiety and loneliness |
| Bragg et al (2013) (Grow2Grow) | Ecominds | Organic dairy farm | England (Kent) | 16 – 25 years | In transition, excluded or recovering from MH problems and in or leaving care |
| Brar-Josan et al (2019) | Cultural brokering in schools | School, home | Canada (West) | 5 – 18 years | CYP in schools with immigrant refugee status, with recognition of trauma and MH issues which may occur alongside this status |
| Brettell et al (2022) | Linking Leeds | Not specified | England (Leeds) | 16 – 25 years | Identified as struggling to cope with stress related to non-medical issues. These issues are predominately linked to social isolation, socioeconomic issues (such as finances, housing, or welfare needs), or have low-medium mental health issues that are creating health inequalities |
| Brown & Jeanneret (2015) | The Evolution Program | Community based dedicated youth arts studio | Australia (Melbourne) | 14 – 25 years | Clinical diagnosis of ARMS. |
| Cale et al (2020) | Get to the Start Line | School | England (East Midlands) | 15 – 16 years | Pupils experiencing stress and anxiety particularly in relation to exams |
| Creamer et al (2020) | The Center | The Center – city hub (non-clinical space) | Canada (small Atlantic city, otherwise not stated) | 16 – 29 years | Mental health issues |
| Children’s Society (2020) | i-Rock | Open access hubs | England (East Sussex) | 14 – 25 years | A range of issues that affect young people from substance use to housing to education, including support for their emotional health. |
| Doari & Mittleman (2021) | Basketball via the Summit Institute in Jerusalem | Summit Institution sports center (community center) | Israel (Jerusalem) | ‘Young adults’ | Psychiatric hospitalisation |
| Donohue et al (2020) | The Optimum Performance Programme in Sports (TOPPS) | Not specified | United States (region not stated) | ‘Youth’ | Being identified as at risk in low income neighbourhoods |
| Drake et al (2021) | Ocean Mind | Beach | Australia (Victoria) | ‘Young people’ | Help-seeking for a mental health problem or identified as being at risk of mental health problems by a mental health professional |
| Efstathopoulou et al (2021) | Arts on Prescription | School, arts classroom | England (East) | 13 – 16 years | Being in receipt of other support services or on a waiting list for other services such as CAMHS. Lack of self-esteem, being vulnerable, self-harm or poor attendance, bullying or difficulty in integration, the student's family situation such as parents' separation, being young carers, poverty and abuse. |
| Godfrey et al (2015) | Wave Project | Beach | England (Cornwall) | 8 – 21 years | Facing mental health issues or social exclusion |
| Gunay & Bacon (2020) | Hip-hop dance programme | Youth Centre in a provincial UK city | United Kingdom (location not stated) | 13 – 25 years, attendance at Youth Centre | Identified as at risk by their school or family due to their behaviour and/or because they were mixing with known offenders |
| Guzder et al (2013) | Dream-A-World (DAW) Project | After school programme | Jamaica (Kingston) | 8 – 9 years | Identified (by teachers) as being at-risk based on poor academic performance, motivation to learn, and behavioural problems |
| Haycock et al (2020) | Tackling the Blues** | School | England (Northwest) | 6 – 16 years | Diagnosed MH issue or displaying behaviours or symptoms associated with poor MH which might lead to diagnosis of mental illness if pupil was accessing specialist MH services. |
| Jones et al (2019) | Tackling the Blues | School | England (Northwest) | 6 – 16 years | Diagnosed MH issue or displaying behaviours or symptoms associated with poor MH which might lead to diagnosis of mental illness if pupil was accessing specialist MH services. |
| Aberdeen Foyer** | | Foyer sites in Aberdeen city and Aberdeenshire | Scotland (Aberdeen) | 16 – 25 years | Housing issues |
| Howe (2007) | Aberdeen Foyer | Foyer sites in Aberdeen city and Aberdeenshire | Scotland (Aberdeen) | 16 – 25 years, already involved in Foyer services | Mood/depression, anxiety/stress, addiction, relationship problems. |
|  | Community Links, Canning Town |  | England (London) | 14 – 25 years | None stated |
|  | Caterpillar service, Barnardo’s Cardiff | Hospital settings and community | Wales (Cardiff) | 12 – 21 years, must have acknowledged mental health problem and be seeking help for this | Emotional difficulties, self-harm behaviours, clinical diagnosis, drug/alcohol misuse, eating problems (complex needs so hard to identify one reason for referral though). |
|  | Support at The Junction, Mind Colchester | Not specified | England (Colchester) | 11 – 18 years, not able to access statutory services | Emotional, behavioural or substance misuse issues. |
|  | The Marketplace | Not specified | England (Leeds) | 13 – 25 years, not able to access statutory services | None stated |
|  | Streetwise | City centre premises, also extensive school outreach | England (Newcastle) | 11 – 25 years | Mental health, sexual health, drug/alcohol misuse |
|  | Icebreak at The Zone | The Zone (youth charity space) | England (Plymouth) | 16 – 25 years | Emotional, behavioural problems such as self-harm, depression, attachment issues, suicidal tendencies, drug/alcohol misuse, eating problems |
| Lederman et al (2019) | Headspace Active** | Youth mental health service | Australia (Sydney) | 14 – 25 years | Clinical diagnosis of ARMS. |
| Rickwood et al (2019) | Headspace | Youth mental health service | Australia (National) | 12 – 25 years | MH issues and at risk of MH issues |
| Manner et al (2021) | Forest School | Local parks and woodland areas nearby but out of sight of the school | Scotland (rural, exact location not stated) | 4 – 13 years, female | Diagnosis of or perception by school/parents/carers of MH concerns such as depression, anxiety, low self-esteem and/or behavioural problems. |
| Marshall et al (2019) | Wave Project | Beach | United Kingdom (Scotland, Scarborough, South Wales, Devon, Cornwall, Dorset, Brighton, Isle of Wight) | 8 – 23 years | Facing or at risk of mental health challenges, social deprivation, or social isolation. |
| Mathias et al (2022) | Foundry | Foundry, a provincial network of integrated youth health and social service centres | Canada (British Columbia) | 12 – 24 years | Physical health, sexual health, mental health and substance use, social services. |
| McIver et al (2018) | WILD (wilderness therapy) | Wilderness area in Victoria | Australia (Victoria) | 18 – 25 years | Recovering from significant MH conditions |
| McKay et al (2012) | Clubhouses – International Center for Clubhouse Development (ICCD) | Clubhouse - physical space in the community | United States (New York and Worcester) | 18 – 25 years | Transitioning from child to adult MH services or with MH issues |
| Montreuil et al (2018) | Leave Out Violence (LOVE) Media Arts Program (MAP) | LOVE head office | Canada (Montreal) | 14 – 17 years | Youths who have been actors, recipients or witnesses of violence. Experience of various types of violence including domestic abuse, rape, sexual harassment, self-harm, bullying, fighting, sexual abuse, gang-related violence, substance abuse and substance-related violence, suicide, and hate crimes. MH issues include depression, suicidal thoughts, eating disorders, and isolation, among others. |
| Parlato et al (1999) | Young Occupations Unlimited (YOU) | Not specified | Australia (Queensland) | 18 – 25 years | Having psychosis, having less than four admissions, having the goal of further education, training or work. |
| Schwan et al (2018) | Arts program | Youth homeless shelter | Canada (region not stated) | 16 – 24 years | Experiencing or at risk of homelessness and associated issues. |
| Stewart et al (2009) | Prodigy Cultural Arts Program | Not specified | United States (Florida) | 10 – 17 years | Involvement in juvenile justice system or at risk in the community |
| Trundle et al (2021) | Phased Model of Adventure Therapy | Not specified | United Kingdom (region not stated) | 8 –16 years | Looked after by the Local Authority and display complex emotional and/or behavioural needs. Complex difficulties including placement in foster care/breakdown of placements, difficulties in education, substance misuse, offending behaviour, high risk of SE, anti-social peer groups, absconding. Behavioural presentations included deficits in attention, physical violence/aggression, self-harm behaviour (previous), low self-esteem, hyperactivity. |
| Tucker et al (2013) | Community-based mental health centre | Community-based mental health center in an urban setting | United States (Mid-West) | 6 – 21 years | Mental health - primarily disruptive disorder or anxiety disorder. |
| Walls et al (2016) | Express Yourself | Not specified | New Zealand (Auckland) | 13 – 24 years | Struggling to cope with a variety of mental health conditions, struggling to cope with relationships, social environments, education or work. |
| Wilson (2017) | The Norfolk Youth Service | Youth Service | England (Norfolk) | 14 – 25 years | Multitude of common problems including health, social, financial and occupational difficulties. Many with psychotic-like experiences in addition to other significant psychopathology (depression, social anxiety) and frequently, significant hx of trauma. |
| Wood et al (2013) | DRUMBEAT | Schools (primary, secondary and 'Intensive English centres' for CYP for whom English not first language) | Australia (region not stated) | Primary and secondary school age | Identified as being at risk of 'negative social and health outcomes including poor school attendance, violence and threats of violence, negative self-image, failing to respond to reasonable requests, experimentation with drugs/alcohol and class disrupting behaviours' as identified by assessment |
| *Active Luton – Life Hacks | | Not specified | England (Luton) | 5 – 19 years | Improving health and wellbeing, social isolation or financial hardship |
| *ActOnIt via Onside Advocacy Worcester | | Not specified | England (Worcester) | Year 6 and above (assume 10+ years), referral must come from school | Low self-esteem, lack of confidence, facing adversity, have caring responsibilities, struggling with home life, difficulty transitioning to high school, low level mental or physical ill health |
| *Barnardo’s Cumbria LINK | | Not specified | England (Cumbria) | 5 – 19 years, must be registered at GP Surgery/attend school in PCNs: Penrith & Eden, Keswick & Solway, Longtown & Brampton | Emotional wellbeing |
| *Battersea Youth Clinic | | Battersea youth clinic | England (London) | Not specified. Registered with PCN. | E.g. loneliness, emotional wellbeing, bullying, family problems, crime |
| *Brandon Centre | | Brandon Centre, school, youth hub, community cafe | England (London) | 16 – 24 years, living, studying or registered with a GP in Camden | Feeling low, anxious, stressed, lonely, isolated, or lacking confidence to make friends, low-to-moderate mental health needs, physical health needs |
| *Chilies | | YMCA Building | England (Barnsley) | 14 – 17 years, living in Barnsley, referrals from GP only | Low level stress/anxiety, education, financial, mental health, housing, bullying and relationship-related issues |
| *HALE Project | | Not specified | England (Yorkshire) | 9 – 19 years, living/accessing services within the Bradford Central District | Loneliness and isolation, improve confidence and self-esteem |
| *Hartlepower | | Not specified | England (Hartlepool) | 11 - 18 years, up to 25 if additional needs | Anxiety, emotional distress, social isolation, excessive worrying, low self-confidence, or combination |
| *Headstart | | Not specified | England (Blackpool, Cornwall, Hull, Kent, Newham, Wolverhampton) | 10 – 16 years | At risk of self-harming or other mental health issues |
| *Healthy London | | In person and online, not specified further | England (Sussex) | 11 – 19 years | Coping with stress and anxiety, sex and relationships, exercise, sleep and long-term conditions |
| *Imago | | Not specified | England (South East and London) | Not specified | Loneliness, stress, money, relationship problems, long-term conditions |
| *Isledon Arts CIC - Lift | | Healthy living hub (Lift) | England (London) | 11 – 25 years | Feeling stressed, overwhelmed or low |
| *Jurassic Coast PCN | | Not specified | England (Dorset) | Not specified: ‘all age service with children and young people’ | Mental and physical health issues, isolation, work/housing/money worries |
| *Kinross High School | | School | Scotland (Kinross) | 5 – 16 years | Support at times of transition, school attendance, barriers to learning, emotional and physical wellbeing |
| *Link Forward | | School or community | England (Bristol) | 7 – 12 years | Any issue impacting on wellbeing e.g. starting a new school, struggling with anxiety, feeling low |
| *Mind the Gap Brighton & Hove | | Home or community | England (Brighton & Hove) | 11 – 25 years | Mental health needs, might be falling between the gaps of services |
| *MYPAS | | Not specified | Scotland (Midlothian) | 12 – 21 years, male | Anxiety, depression, bereavement, loneliness, relationship problems, body image, anger |
| *New Forest PCN | | Not specified | England (New Forest) | 11 – 25 years | Emotional health and wellbeing issues - MH, relationships, isolation, money/housing (16+), school and education issues, accessing groups in your local area |
| *No Limits | | Emergency department and community | England (Southampton, Hampshire, Portsmouth, Isle of Wight) | 11 – 26 years | Attending ED in crisis (violent crime or MH) |
| *No Limits Southampton | | Not specified | England (Southampton) | 13 – 25 years | Emotional health and wellbeing issues |
| *Safety Nets – Yorkshire Sport | | Local football club | England (Yorkshire) | Up to age 18 years | On CAMHS waiting list |
| *SOFEA – Mental Wealth Academy | | Not specified | England (Oxford, Milton Keynes) | 16 – 25 years | None stated |
| *Stort Valley Healthcare | | Includes online, not further specified | England (Hertfordshire & Essex) | 11 – 25 years | Mental health conditions and general wellbeing issues |
| *St Stephen’s Primary School | | School | England (Surrey) | 5 – 11 years | Experiencing transition, parental separation/bereavement, behavioural issues, attendance issues, lack of confidence, low self-esteem, family issues |
| *Three Rivers Academy | | School | England (Surrey) | 11 – 16 years | MH, behavioural, school, family or financial issues |
| *Thrive | | Drop in café | England (Rochdale) | Not specified | Diet, exercise, general lifestyle |
| *Waverly Abbey Junior School | | School, home | England (Surrey) | 5 – 11 years | School attendance, family issues, emotional support |
| *Wellbeing Exeter | | Not specified | England (Exeter) | 11 – 18 years, living in Exeter | Loneliness and anxiety |
| *Well Centre Lambeth | | The Well Centre | England (London) | 11 – 20 years, not in urgent care or tier 3 CAMHS | None stated |
| *Young People’s Social Prescribing North Cotswolds | | Not specified | England (Gloucestershire) | 6 – 19 years, living in North Cotswolds and registered at specific GP Surgeries | MH and wellbeing e.g. exam worries, problems at school or college, careers guidance, mental health, friendships |
| *Youth Link | | Watford football club | England (West Hertfordshire) | 10 – 24 years, registered with GP in West Herts area | Physical and emotional health and wellbeing, e.g. isolation, persistent low mood, health condition, advice on personal situation, carer responsibilities |
| *Zone West Newcastle | | Not specified | England (Newcastle) | Not specified | None stated |

**grey literature sources*

**** *Where there is a reference to the same site but in a separate study, references have been organised to group studies pertaining to the same site together.*
