# Supplementary material for: The use of social prescribing and community-based wellbeing activities as a potential prevention and early intervention pathway to improve adolescent emotional and social development: a systematic mapping review
Source: BMC Public Health. 2025 Oct 15;25:3495. doi: 10.1186/s12889-025-24413-5 (PMC12522731; doi:10.1186/s12889-025-24413-5)
Supplement: Supplementary file 4 — Supplementary Material 4. [file 12889_2025_24413_MOESM4_ESM.docx]

**Table 3. Synthesis of pathway data from included papers**

| **Author/year** | **Service** | **Referrer(s)** | **Linking function** | **Length of pathway/support** | **Activity or support pillar** | | | | | | **Notes** |
| --- | --- | --- | --- | --- | --- | --- | --- | --- | --- | --- | --- |
|  |  |  |  |  | Advice & information | Arts & heritage | Natural  Environment | Physical activity | Other social  Support | Other specific  support or  activity |  |
| Appelqvist-Schmidlechner et al (2021) | Icehearts | Pre-school personnel, social workers, parents | Mentor providing sports and support at school, after school and at home | Up to 12 years |  |  |  | ✓ |  | ✓ |  |
| Bertotti et al (2020) | Young People Social Prescribing (YPSP) pilot - Sheffield site | Friend/family, GP, school/college, MH service, adult social care/services, internal, project staff/volunteer, self-referral, community hospital, other | Link Worker | 4-5 sessions over 2-3 months | ✓ |  |  |  | ✓ |  | Volunteering |
|  | Young People Social Prescribing (YPSP) pilot - Brighton & Hove site | Friend/family, GP, school/college, MH service | Link Worker | 4-5 (maximum 8) one-hour sessions for up to 12 months | ✓ |  |  |  | ✓ |  | Sussex Night Stop |
|  | Young People Social Prescribing (YPSP) pilot - Luton site | Friend/family, MH service, adult social care/services, self-referral | Link Worker | 40-minute sessions for up to 12 weeks | ✓ | ✓ |  | ✓ | ✓ |  | Gaming; youth services |
| Bragg et al (2013) | Ecominds | GPs, community mental health services, leaving care services, CYP services and Connexions | None | Six-week introductory programme then up to 3 days per week for up to 2 years |  |  | ✓ |  |  |  |  |
| Brar-Josan et al (2019) | Cultural brokering in schools | Schools | Cultural broker | Not stated |  |  |  |  |  |  | Educational cultural brokers in schools linking to MH support |
| Brettell et al (2022) | Linking Leeds | GP, self-referral, or introduction from an alternative professional | None | Not stated | ✓ |  |  |  | ✓ |  |  |
| Brown & Jeanneret (2015) | The Evolution Program | Not stated | Youth Worker | 8-week programme |  | ✓ |  |  |  |  |  |
| Cale et al (2020) | Get to the Start Line | Teachers/school staff | School champion | Six 1.5-hour workshops |  |  |  | ✓ |  |  |  |
| Creamer et al (2020) | Center | Self-referral (also formal referral from professionals but unspecified) | None | Not stated | ✓ | ✓ |  | ✓ | ✓ |  |  |
| Children's Society (2020) | Open access hubs | Self-referral | 'Service staff' | Not stated | ✓ |  |  |  |  |  | Local voluntary sector providers, education providers |
| Doari & Mittleman (2021) | Basketball via the Summit Institute in Jerusalem | Mental health professionals | None | Not stated |  |  |  | ✓ |  |  |  |
| Donohue et al (2020) | The Optimum Performance Programme in Sports (TOPPS) | Designated administrator, coach, identified youth, parent, teacher, school | None | Not stated |  |  |  | ✓ |  |  |  |
| Drake et al (2021) | Ocean Mind | Mental health professional, GP | Mentor (unclear if linking role) | Six-week programme |  |  | ✓ | ✓ |  |  |  |
| Efstathopoulou et al (2021) | Arts on Prescription | School staff members – individual or team (incl. pastoral team/staff members with MH/SEN role) | School staff (internal) | 10 x weekly 2-hour workshops |  | ✓ |  |  |  |  |  |
| Godfrey et al (2015) | Wave Project** | Schools, NHS healthcare providers (GPs, nurses, psychologists), social and family services, other child support agencies (e.g. Action for Children, Young carers) | None | Six weeks. CYP can continue with surf club after this to become volunteer helpers/surf-mentors |  |  | ✓ | ✓ |  |  |  |
| Marshall et al (2019) | Wave Project | CAMHS, social/foster care**,** schools, mental health charities/organisations | None | Six-week programme, weekly 2-3 hours sessions. CYP can then opt-in to surf club that provides a continuation of the intervention |  |  | ✓ | ✓ |  |  |  |
| Gunay & Bacon (2020) | Hip-hop dance programme | Youth centre support workers, schools, self-referral | Mentor (unclear if linking role) | 3-4 two-hour sessions per week (after school) plus 1:1 mentor sessions once a week |  | ✓ |  | ✓ |  |  |  |
| Guzder et al (2013) | Dream-A-World (DAW) Project | School teachers | None | Seven semesters of an after-school programme or 40-hour summer workshops (4 days/wk for three weeks) |  | ✓ |  |  |  |  |  |
| Haycock et al (2020) | Tackling the Blues** | Schools | School staff | Weekly one-hour sessions, average attendance of 25 sessions |  |  |  | ✓ |  |  |  |
| Jones et al (2019) | Tackling the Blues | Schools | Not stated | One school year |  |  |  | ✓ |  |  |  |
| Aberdeen Foyer** | | Aberdeen City Council Housing Advice Service and Homelessness Service | Unclear | Not stated | ✓ |  |  |  |  |  |  |
| Howe (2007) | Aberdeen Foyer | Self-referrals (from CYP already using Foyer services), referrals from Foyer support workers | None | Not stated | ✓ |  |  |  |  |  |  |
|  | Community Links | All professionals, families, friends, self-referrals, internal referrals within Community links | None | Not stated |  | ✓ |  | ✓ |  |  |  |
|  | Caterpillar service | Referrals accepted from any source including voluntary and statutory agencies, friends, family members, self-referral | Project worker | Once a week |  |  |  |  |  |  | Not specified |
|  | Support at the Junction | Education institutions, youth judiciary system, A&E departments, GPs, youth services, social services, local primary care trust, mental health trusts, drug and alcohol services | Workers from the Junction | Not stated |  |  |  |  |  |  | Not specified |
|  | The Marketplace | Self-referrals, parents, friends, statutory services including GP and CAMHS, internal referrals from within the Marketplace | None | Not stated |  |  |  |  |  |  | Not specified |
|  | Streetwise | Voluntary or statutory agencies, families, carers, or self-referral | None | Not stated | ✓ |  |  |  |  |  | Support based around mental health; sexual health; and drug and alcohol misuse - counselling/education |
|  | Icebreak at The Zone | Professional agencies, friends, family, self-referral, within service referral | None | Not stated |  |  |  |  | ✓ |  | As above |
| Lederman et al (2019) | Headspace Active** | Primary care physician, Headspace within-service referral by clinicians | Within-service referral by clinicians | Twelve-week programme, minimum of five sessions |  |  |  | ✓ |  |  |  |
| Rickwood et al (2019) | Headspace | All types of referral including self-referral and schools, referrals within service and to external | Headspace links CYP to other services or in-house | Not stated | ✓ |  |  |  |  |  | Very general: *After the holistic needs of each young person are identified, they are met through an integrated care pathway with a coordinated approach to the mix of services required* |
| Manner et al (2021) | Forest School | Teachers, parent/carer | School links to activity | Weekly sessions of 3 hours over 12 weeks |  |  | ✓ | ✓ |  |  |  |
| Mathias et al (2022) | Foundry | Peers, family, GPs, school counsellors, internet | None | Not stated | ✓ |  |  |  | ✓ |  |  |
| McIver et al (2018) | WILD (wilderness therapy) | Referral from within resident’s programme | Key worker | Nine to eleven sessions within a 12 month residential bush therapy programme |  |  | ✓ | ✓ |  |  |  |
| McKay et al (2012) | Clubhouses – International Center for Clubhouse Development (ICCD) | Hospitals or other treatment programmes (e.g. outpatient clinics, continuing day treatment, integrate treatment and recovery programmes, vocational rehabilitation etc.), homeless shelters, schools, existing members, mental health departments | Clubhouse staff support referrals | Not stated | ✓ |  |  |  |  |  | Outreach to other organisations |
| Montreuil et al (2018) | Leave Out Violence (LOVE) Media Arts Program (MAP) | Social services, school teachers, guidance counsellors | None | Weekly two-hour sessions |  | ✓ |  |  |  |  |  |
| Parlato et al (1999) | Young Occupations Unlimited | Acute inpatient unit, community mental health teams, private psychiatrists, GPs, non-government organisations e.g. Schizophrenia Fellowship, self-referral, family | None | No duration stated: Discharge occurs when the YP either successfully undertakes paid employment, voluntary work or further education/training, or expresses a wish to be no longer involved | ✓ |  |  |  |  |  |  |
| Schwan et al (2018) | Arts program | Internal and external referrals from professionals (no self-referral) | None | Not stated |  | ✓ |  |  |  |  |  |
| Stewart et al (2009) | Prodigy Cultural Arts Program | State juvenile justice system, does not state how those referred in who were not from justice system get referred in** | None | Eight-week programme, 3 hours per week |  | ✓ |  |  |  |  |  |
| Trundle et al (2021) | Phased Model of Adventure Therapy | Local authorities | None | Residential programme between 2 and 6 weeks |  |  | ✓ | ✓ |  |  |  |
| Tucker et al (2013) | Community-based mental health centre | Schools, physicians, case workers (if involved in child services) | None | Most AT groups were open-ended. Two hours, once per week |  |  | ✓ | ✓ |  |  |  |
| Walls et al (2016) | Express Yourself | Community mental health, addictions services, self-referral | Youth mental health/addictions workers | Not stated | ✓ | ✓ |  |  |  |  |  |
| Wilson (2017) | The Norfolk Youth Service | Any source including self-referrals | Youth service | Not stated | ✓ |  |  |  |  |  | Local partner agencies, voluntary sector agencies |
| Wood et al (2013) | DRUMBEAT | School staff | None | Ten-week programme |  | ✓ |  |  |  |  |  |
| *Active Luton - Life Hacks | | Any professional for ages 5-19, certain GP Surgeries in Bedford, Central Beds or Milton Keynes for ages 11-18 | Link Worker | LW support up to 12 weeks | ✓ | ✓ |  | ✓ | ✓ |  |  |
| *ActOnIt via Onside Advocacy | | School | 1:1 Support Team | Not stated |  |  |  |  | ✓ |  | Supported participation in activities |
| *Barnardo's Cumbria LINK | | GP, other healthcare professional, school professional | Link Worker | Not stated |  |  |  |  |  |  | Community engagement |
| *Battersea Youth Clinic | | GP/self-referral (via online form to book appointment with youth link worker) | Youth Link Worker | Not stated | ✓ |  |  | ✓ | ✓ |  | Mentoring - social support |
| *Brandon Centre | | Professionals via referral form | Wellbeing Link Workers (Social Prescribers) | Up to six LW sessions |  | ✓ |  | ✓ | ✓ |  | 'And much more' including fashion and beauty, cooking and baking |
| *Chilypep | | GPs only | Unclear - customised care plan with regular support | 12 weeks | ✓ |  |  |  |  |  | Referral reasons include mood, education, financial, housing, relationship issues but activities not stated |
| *HALE Project | | Schools, colleges, alternative education settings, GPs, care/residential homes, specialised YP services, youth clubs, self-referral | Unclear - 1:1 tailored support | Not stated |  |  |  |  |  |  | Activities and services |
| *Hartlepower | | Schools, parents, social workers, self-referral (can be other) | Social Prescribing Link Worker | Led by young person |  |  |  |  |  |  | Not specified - led by YP |
| *Headstart | | GP or any other professional | Youth and Community Facilitators | 1-12 sessions of support |  | ✓ | ✓ | ✓ | ✓ | ✓ |  |
| *Healthy London | | GP, self-referral | Link Worker/ Community Navigator | Not stated |  |  |  |  |  |  | No specific activities: *Being Well Salford uses social prescription pads for GPs to write paper ‘prescriptions’ to be collected by a community organisation, who gets in touch with people directly to discuss support and to be linked up with suitable community-based resources* |
| *Imago | | GP | Social Prescribing Link Coordinator | Not stated | ✓ |  | ✓ | ✓ | ✓ |  |  |
| *Isledon Arts CIC - Lift | | Via participating partners: COLA Highgate Hill, Paediatric Rheumatology (Whittington), Paediatric Diabetes (Whittington), Healthy Living Service (Brandon Centre), School Wellbeing Service, GPs | Link Workers | Initial plan for 12 weeks then review: depends on YP |  | ✓ | ✓ | ✓ | ✓ | ✓ | Other includes e.g. cooking, bike maintenance |
| *Jurassic Coast PCN | | GP, nursing, admin team, community groups, statutory services, self-referral | Link Workers (PCN) | Not stated | ✓ |  |  |  | ✓ |  | Assistance in becoming more involved in your community |
| *Kinross High School | | Primary School or secondary House Teams | Community Link Worker | Not stated |  |  |  |  |  |  | No detail - just talks about signposting and working collaboratively with community services |
| *Link Forward | | GPs or schools | Link Workers (PCN) | Up to 6 session plus check-in with parent/carer |  | ✓ |  |  |  |  | Sometimes signposting to parent support, sometimes linking to services that can help with wellbeing |
| *Mind the Gap Brighton & Hove | | GPs | Link Worker | Not stated |  |  |  | ✓ | ✓ |  | 'Wellbeing services' |
| *MYPAS | | Not stated | Young Men's Workers | Not stated |  | ✓ | ✓ | ✓ |  | ✓ |  |
| *New Forest PCN | | GP or self-referral via website/email | Social Prescriber | Not stated | ✓ |  |  |  | ✓ | ✓ | Not specific - MH, relationships, isolation, money/housing (16+), school and education issues, accessing groups in your local area |
| *No Limits | | Emergency department | ED Youth Workers | Not stated |  |  |  |  |  |  | Not specified: *connecting them with specialist support and other community services through Social Prescribing* |
| *No Limits Southampton | | Unclear, suggests self-referral | Social Prescription Worker | No maximum offer |  | ✓ |  | ✓ | ✓ |  | Volunteering |
| *Safety Nets - Yorkshire Sport | | CAMHS waiting list | None | Weekly for two hours after school across 8 weeks |  |  |  | ✓ |  |  |  |
| *SOFEA | | Not stated | Link Workers (PCN) | Not stated |  |  |  |  |  |  | No specific activities |
| *Stort Valley Healthcare | | GP | Young Person's Social Prescriber & Link Worker (PCN) | Not stated | ✓ | ✓ |  |  | ✓ |  |  |
| *St Stephen's Primary School | | Parents | Home School Link Worker | Not stated | ✓ |  |  |  |  |  |  |
| *Three Rivers Academy | | Parent, teacher, self-referral | Home School Link Worker | Not stated | ✓ |  |  |  |  |  |  |
| *Thrive | | Self-referral, GP, CAMHS, schools | Not stated: drop-in café | Not stated | ✓ | ✓ |  | ✓ |  |  |  |
| *Waverly Abbey Junior School | | Parents | Home School Link Worker | Not stated |  |  |  |  |  |  | Not specific: *Helping families and children to access services, resources and local community information.* |
| *Wellbeing Exeter | | GP, Self-referral, Parents, Schools | Link Worker, Community Connectors | 6-8 sessions (1 per week), open-door policy following completion of sessions | ✓ | ✓ |  | ✓ |  |  |  |
| *Well Centre Lambeth | | Self-referral, by another person or professional | Health & Wellbeing Practitioners (assessed by GP first) | Up to 3 months with H&WP | ✓ | ✓ |  | ✓ | ✓ |  | Mentoring under 'other social support' |
| *Young People's Social Precsribing North Cotswolds | | GP | Social Prescribers | Not stated |  |  |  |  |  |  | No specifics |
| *Youth Link | | GP, teacher, other service, health provider, youth mentor or worker and self/parent referral via form | Not stated (via Youth Link Action Plan) | Six meetings (face-to-face/Zoom/phone) plus check-ins and reviews | ✓ |  |  | ✓ | ✓ |  | Volunteering, health support |
| *Zone West Newcastle | | **Schools, GPs** | Link Workers (with and in schools) | Weekly LW meetings but duration not stated |  |  |  |  |  |  | Just states 'linking them to activities' |

**grey literature sources*

*** Where there is a reference to the same site but in a separate study, references have been organised to group studies pertaining to the same site together.*
